# Physical activity, sedentary behaviour, and sleep knowledge and self-efficacy among parents of young children in Canada

**DOI:** 10.1186/s44167-024-00051-x

**Published:** 2024-05-17

**Authors:** Brianne A. Bruijns, Matthew Bourke, Kendall Saravanamuttoo, Patricia Tucker

**Affiliations:** 1 School of Occupational Therapy, Faculty of Health Sciences, Western University, 1201 Western Road, Elborn College, Room 2547, London, ON N6G 1H1 Canada; 2 Health and Wellbeing Centre for Research Innovation, The University of Queensland, Brisbane, QLD Australia; 3 Children’s Health Research Institute, London, ON Canada

**Keywords:** Movement behaviours, Early childhood, Parental influences, Knowledge, Self-efficacy

## Abstract

**Background:**

Engaging in healthy movement behaviours in early childhood is beneficial for children’s development, and parents play a critical role in shaping movement habits during these formative years. This study aimed to explore parents’ knowledge of the *Canadian 24-Hour Movement Guidelines for the Early Years* (The Guidelines) and their perceived knowledge and self-efficacy of movement behaviour concepts in early childhood. The influence of sociodemographic characteristics on these variables was also examined.

**Methods:**

Via a cross-sectional online survey with parents/guardians (*n* = 576) of young children in Canada, participants’ knowledge of The Guidelines (11 items), and perceived knowledge (11 items; 5-point scale) and self-efficacy (11 items; 11-point scale) of movement behaviour concepts in early childhood were examined. Descriptive statistics were calculated for independent variables and multivariate regression models were estimated to examine sociodemographic factors that were associated with participants’ knowledge and self-efficacy.

**Results:**

Few participants (11.9%) reported to be familiar with The Guidelines. Parents were most knowledgeable about the screen time guideline for children under 2 years (75.0% correctly identified the guideline) and least familiar with the recommendation for toddler/preschooler total physical activity (14.7% correctly identified the guideline). Parents/guardians perceived they were the most knowledgeable about safe sleep practices (65.9% very/extremely knowledgeable) and least knowledgeable about muscle- and bone-strengthening activities (71.8% not at all/somewhat knowledgeable). Overall, parents/guardians were moderately confident in promoting healthy movement behaviours (*M* = 6.01; *SD* = 1.73). Participants with higher levels of education reported significantly higher perceived knowledge compared to those who were less educated (*p* = .004), and parents/guardians identifying as White reported significantly higher levels of self-efficacy compared to parents/guardians from minority ethnic groups (*p* = .028). Finally, parents/guardians who were more physically active reported both higher perceived knowledge (*p* < .001) and self-efficacy (*p* < .001) than those who were less active.

**Conclusion:**

These findings shed light on the need to raise awareness of The Guidelines among parents of young children and highlight specific movement behaviour concepts in need of focus in future training for this population. Targeted education for parents/guardians from lower socioeconomic groups is also needed to address equity concerns.

**Supplementary Information:**

The online version contains supplementary material available at 10.1186/s44167-024-00051-x.

## Background

Engaging in healthy movement behaviours (i.e., physical activity, sedentary behaviour, sleep) is critical for young children’s (0–4 years) development and well-being [1, 2]. Evidence highlights the importance of these movement behaviours in combination across the whole day – rather than looking at them as individual entities – to collectively support young children’s health and well-being [3]. The interrelationship among movement behaviours has informed the development of *The Canadian 24-Hour Movement Guidelines for the Early Years (0–4 years)* [4], which provide integrated recommendations for healthy physical activity, sedentary behaviour, and sleep for infants (< 1 year), toddlers (1–2 years), and preschoolers (3–4 years). These guidelines have more recently been adopted in several other countries including South Africa [5] and Australia [6], as well as by the World Health Organization [7]. Briefly, a healthy 24 hours for infants includes interactive floor-based play several times, including at least 30 minutes of tummy time for those not yet mobile; 12 to 17 hours of high-quality sleep depending on age; no sedentary screen time; and, limiting periods of restraint (e.g., in stroller or highchair) to no more than 1 hour per day. Toddler and preschooler recommendations suggest that children engage in 180 daily minutes of physical activity (1 hour of which should be at a moderate-to vigorous-intensity for preschoolers); no more than 1 hour of prolonged sitting; no screen time under 2 years, and no more than 1 hour for those 2 years and older; and between 10 and 14 hours of good quality sleep depending on age [4]. Evidence from a recent systematic review and meta-analysis across 23 countries suggests that only 11% of young children are meeting the overall movement behaviour guidelines [8]. This is concerning, as meeting each component of the guidelines is associated with many physical (e.g., bone health), psychosocial (e.g., health related quality of life), and cognitive (e.g., behavioural) benefits [9]. Thus, it is critical to implement public health strategies that target young children’s adherence to the guidelines to ensure lifelong health outcomes.

Research has found that children’s adherence to movement guidelines can also vary by a variety of sociodemographic factors [8]. For example, in a study of preschoolers’ (*n* = 107) movement behaviours, Kracht et al. [10] found that African American preschoolers and children who lived at or below the poverty line were less likely to meet the individual sleep and sedentary behaviour guidelines, as well as all three combined movement behaviour guidelines, compared with those from other ethnicities or income levels (*p* < .01). Similarly, a study among parents of children (5 to 17 years) in Canada (*n* = 1,208) found that fathers had lower support of the 24-hour movement guidelines than mothers [11], indicating that they may be less engaged in their children’s movement behaviours. Further, a Singaporean study found that pre-pregnancy maternal physical activity and television viewing time were the only significant predictors of children’s (*n* = 547) adherence to integrated 24-hour movement behaviour guidelines at 5.5 years of age [12]. While a preliminary association is emerging, reiterating the importance of sociodemographic factors with respect to movement behaviour promotion, more research is needed to determine if such factors mediate the effect of parental knowledge and self-efficacy on movement behaviour promotion among young children.

Investigating parents’ and guardians’ role in supporting children’s health is imperative, as they are often considered facilitators for their children’s movement behaviours through mechanisms including encouragement, support, role modelling, and their perceived confidence and self-efficacy to promote these behaviours [13, 14]. During a 24-hour period, parents and guardians are responsible for setting bedtime routines [15], monitoring screen time and access to electronic devices [16], and providing physical activity and active play opportunities [17]. Parental movement behaviours are also known to have a direct impact on children’s movement behaviours, with studies finding significant dyadic correlations between parents’ and their child’s physical activity and screen time [18]. Yet, many parents have reported they lack the knowledge and skills to promote their children’s healthy movement behaviours [19], which may be problematic given their influence over their child’s engagement in healthy movement behaviours.

As a result of the knowledge gaps amongst many parents and guardians with young children, governing bodies such as the World Health Organization [20] and Canada’s Sport for Life [21] have called attention to the need for increased parental education, including the implementation of appropriate capacity building initiatives. Specifically, there is strong evidence of the effectiveness of cognitive-behavioural approaches to support parents’ knowledge, skills, and self-efficacy to promote healthy movement behaviours [22]. A critical component of parents’ and guardians’ behaviour change is self-efficacy – or one’s confidence to execute a particular behaviour – and is based in Bandura’s Social Cognitive Theory (SCT) [23]. This construct is gaining attention in the field, with a multitude of studies supporting the relationship between parental self-efficacy and children’s physical activity and media use [24–26]. For instance, a study conducted by Kieslinger et al. [24] that explored the impact of a health promotion program on German parents’ self-efficacy to promote their kindergarteners’ physical activity found that higher parental self-efficacy was associated with significantly more daily physical activity amongst children than parents with lower self-efficacy. Similarly, a longitudinal Australian study found that higher maternal self-efficacy was associated with lower television viewing in young children [27]. Therefore, it is important to consider parental self-efficacy as a mechanism to supporting young children’s healthy movement behaviours.

Currently, little is known about parents’ and guardians’ perceived knowledge and self-efficacy to facilitate a variety of healthy movement behaviour components (e.g., the guidelines, fundamental movement skills, outdoor play opportunities). This is important to understand, as fostering knowledge acquisition facilitates increases in self-efficacy (per the SCT). Therefore, such insights can inform educational resource development and training for parents regarding healthy movement behaviours in the early years. As such, the **M**ovement **E**ducation for parents of **YOU**ng children (**ME & YOU**) needs assessment study was conducted to explore parents’ and guardians’ educational background regarding promoting healthy movement behaviours in early childhood (findings reported in Bruijns et al. [28]) and to examine their knowledge and self-efficacy as they relate to promoting healthy movement behaviours. This manuscript will present findings from the latter objective, and specifically aimed to: (1) explore parents of young children’s knowledge of the *Canadian 24-Hour Movement Behaviour Guidelines for the Early Years*, as well as their perceived knowledge and self-efficacy of movement behaviour-related concepts in early childhood; and, (2) examine if parents’ knowledge of the 24-hour movement behaviour guidelines, perceived knowledge relating to physical activity, screen time, and sleep practices, and/or self-efficacy ratings were influenced by sociodemographic characteristics, their personal physical activity and screen time habits, or the movement behaviour education they received in their prenatal, postnatal, or pediatric care. To our knowledge, this is the first study to provide a fulsome understanding of parents’ and guardians’ knowledge and self-efficacy related to their young children’s movement behaviours and will be used to inform educational material and training for parents/guardians in Canada.

## Methods

A cross-sectional study design was employed for the ME & YOU study, which was approved by the Non-Medical Research Ethics Board at Western University (REB# 122,865).

### Study procedures and participant recruitment

Parents/guardians living in Canada with at least one child under the age of 5 years were recruited via social media advertisements (e.g., Twitter, Instagram) and recruitment flyers distributed via email to members of various parenting/family organizations from May to August 2023. Implied consent was given by commencing the survey.

#### Online survey

An online survey (*n* = 57 items; Appendix A) was developed and administered in English and French via Qualtrics for the purposes of this study. Eleven items were used to test parents’ knowledge of the *Canadian 24-Hour Movement Guidelines for the Early Years*. Parents were presented with multiple choice questions and were asked to select the correct guideline (e.g., “How many minutes of screen time should a 1-year-old be limited to each day?”). Parents were also asked 11 questions to rate their perceived knowledge related to physical activity, sedentary time, and sleep related practices (e.g., “Fundamental movement skill [e.g., jumping, throwing, balancing] development”; Cronbach’s alpha = 0.92). A modified version of the *Early Childhood Educator Confidence in Outdoor Movement, Physical Activity, and Sedentary and Screen behaviours* (ECE-COMPASS) questionnaire [29] was used to measure participants’ self-efficacy on an 11-point Likert-Scale (Cronbach’s alpha = 0.92). The original version the ECE-COMPASS tool was developed to assess self-efficacy among early childhood educators relating to physical activity, sedentary behaviour, and outdoor play. Modifications were made to the wording of selected questionnaire items (i.e., items 2, 3, 10, 13, 15–17, 19–21) to assess parents’ perceived self-efficacy instead of early childhood educators’ (e.g., “*Avoid screen-based technology during childcare hours*” was modified to “*Adhere to age-appropriate recommendations for screen time among my children [i.e., no screen time < 2 years, maximum 1hr/day 2–4 years]*”). Further, one new item was adopted to assess parents’ self-efficacy related to sleep behaviours (“*Support my child(ren) in meeting age-appropriate sleep recommendations [i.e., 14-17hrs/day < 4 months; 12-16hrs/day 4–11 months; 11-14hrs/day 1–2 years; 10-13hrs/day 3–4 years]*”).

Participant demographics (*n* = 15 items) were also gathered, including their: age; gender; racial background; province/territory of residence; family situation (i.e., single-parent, double-parent, guardian-led, other); number and ages of their child(ren); the care arrangement for their child(ren); personal levels of physical activity and recreational screen time; highest level of education; employment status; annual household income; and, housing type. Additional survey items included: (1) whether participants received education about movement behaviours in early childhood during their pre-/postnatal care or child’s doctor/pediatrician appointments (*n* = 3 items); (2) if they sourced their own information about these topics (e.g., online, via apps, asking family or friends; *n* = 1 item); (3) participants’ perspectives about movement behaviour content areas they would like to learn more about (*n* = 4 items); and, (4) the format they would like to use to access additional information (*n* = 1 item). These results are presented elsewhere [28].

### Data Analysis

All data analysis was conducted in SPSS (version 29). To determine parents’ knowledge and self-efficacy related to young children’s movement behaviours, frequencies were calculated for the number of parents who correctly identified the 24-hour movement behaviour guidelines for young children and perceived knowledge, while means (*M*) and standard deviations (*SD*s) were calculated for self-efficacy ratings. Multivariate linear regression models were estimated to examine sociodemographic variables that were independently associated with knowledge of the *Canadian 24-hour Movement Guidelines for the Early Years*, and perceived knowledge and self-efficacy of movement behaviour concepts in early childhood. Due to the exploratory nature of this study, as well as the single hypothesis that sociodemographic factors are related to the outcomes identified, no adjustments for multiple comparisons were made [30]. In order to increase cell size and improve the power of the analysis, the pragmatic decision was also made to combine non-White ethnicities into a single “ethnic minority” group. Prior to estimating the linear regression model, a missing value analysis was conducted to determine patterns of missingness; results demonstrated that data was missing completely at random (Little’s MCAR x2[237] = 237.91, *p* = .544). Therefore, cases with missing values were deleted listwise, and only participants with valid data for all independent variables were included in the multivariate analyses (*n* = 350).

## Results

### Participant demographics

This study sample included 576 Canadian parents/guardians of children under 5 years of age. Parents/guardians were 34.4 years old (*SD* = 3.8 years), and the majority were Caucasian (76.7%), female (94.2%), from double-parent households (95.4%), and from Ontario (33.8%), British Columbia (16.1%), or Québec (12.1%). Just over half (53.6%) of parents/guardians had one child, 34.1% had two children, and 12.3% had three or more children; age ranges for those in the early years were fairly evenly split, with 39.0% having an infant, 52.4% having a toddler, and 47.0% having a preschooler. Only 22.7% of parents/guardians reported to meet the adult physical activity guideline (i.e., 150 min of moderate-to-vigorous physical activity per week), while the majority (77.3%) reported to meet the recreational screen time guideline (i.e., no more than 3 h per day) in the *Canadian 24-Hour Movement Guidelines for Adults (18–65 years)* [31]. See Bruijns et al. [28] for complete participant demographics and Appendix B for demographics of parents/guardians included in the multivariate analyses (*n* = 350).

### Knowledge

#### Knowledge of the Canadian 24-Hour Movement Guidelines for the Early Years

A total of 65 participants (11.9%) reported they were familiar with the *Canadian 24-Hour Movement Guidelines for the Early Years*; of those who stated they were familiar with these guidelines, more than half (51.6%) indicated they were only somewhat familiar with them. When asked about their knowledge of the 24-hour movement behaviour guidelines, parents were able to correctly identify recommendations pertaining to sleep most consistently, with 49.2%, 50.5%, and 47.1% of parents/guardians selecting the correct response for infants’ (4–11 months), toddlers’, and preschoolers’ sleep time recommendations, respectively. While the majority of participants (75.0%) correctly identified the screen time guideline for 0-2-year-olds (i.e., 0 minutes per day), only 22.3% selected the correct response for the recommended screen time limit for those 2 to 4 years old (i.e., 60 minutes per day). The physical activity-related guideline questions were the most poorly known by parents, with only 35.3%, 14.7%, and 20.4% of respondents answering correctly for the infant tummy time, toddler/preschooler total physical activity, and the preschooler moderate-to-vigorous physical activity recommendations, respectively.

Parents’ and guardians’ knowledge of the *Canadian 24-Hour Movement Guidelines for the Early Years* was associated with their family situation (*p* = .028) and employment status (*p* = .013; Table 1). Specifically, participants in double parent households answered more questions about the guidelines correctly compared to those from other family arrangements (MD = 1.42, 95% CI = 0.16, 2.68, *p* = .027). Considering employment status, participants on parental leave answered significantly fewer questions pertaining to the guidelines correctly compared to parents who worked full-time (MD = -0.82, 95% CI = -1.30, -0.34, *p* < .001) and those employed in occasional/support positions (MD = -0.91, 95% CI = -1.82, -0.00, *p* = .050). Associations between each of the independent variables with knowledge of the *Canadian 24-Hour Movement Guidelines for the Early Years* are displayed in Appendix C.

**Table 1:** ANOVA results from regression models testing associations with knowledge of the Canadian 24-hour movement guidelines for the early years and perceived knowledge and self-efficacy of movement behaviour concepts

Sociodemographic Variable		Knowledge of Guidelines		Perceived Knowledge		Self-Efficacy	
	df	*F*	*p*	*F*	*p*	*F*	*p*
Age	1	0.19	0.668	2.26	0.134	0.38	0.537
Sex	1	1.56	0.212	0.03	0.856	0.15	0.703
Ethnicity	1	0.05	0.820	0.43	0.514	4.89	0.028*
Province/Territory	6	1.24	0.284	0.51	0.802	0.45	0.848
Family situation	1	4.95	0.028	2.26	0.133	0.12	0.727
Number of children cared for	2	1.76	0.174	0.53	0.590	0.46	0.635
Highest level of education	3	0.51	0.677	4.47	0.004*	0.43	0.734
Employment status	5	2.94	0.013*	2.10	0.066	2.65	0.023*
Annual household income	3	1.90	0.130	1.15	0.328	2.17	0.092
Housing type	5	0.37	0.867	0.82	0.534	2.00	0.078
Physical activity guidelines	1	0.10	0.757	12.655	< 0.001*	41.32	< 0.001*
Screen time guidelines	1	0.31	0.578	0.32	0.575	2.99	0.085
Survey language	1	0.00	0.959	0.10	0.747	0.05	0.829
Prenatal physical activity education	1	1.14	0.287	0.05	0.817	0.50	0.479
Prenatal sedentary education	1	1.12	0.291	0.15	0.697	0.20	0.652
Prenatal sleep education	1	2.59	0.108	0.22	0.882	0.03	0.864
Postnatal physical activity education	1	0.57	0.453	1.73	0.190	0.18	0.673
Postnatal sedentary education	1	3.79	0.052	0.16	0.691	0.27	0.604
Postnatal sleep education	1	0.05	0.829	0.06	0.815	1.70	0.193
Pediatric physical activity education	1	0.24	0.625	0.02	0.896	0.98	0.323
Pediatric sedentary education	1	1.90	0.166	0.80	0.371	0.34	0.561
Pediatric sleep education	1	0.18	0.675	0.55	0.457	0.71	0.791

Note. * = *p* < .05.

#### Perceived knowledge of movement behaviour concepts in early childhood

Participants were most knowledgeable about safe sleep practices (65.9% reported to be very or extremely knowledgeable), followed by the health benefits of outdoor play (50.1% reported to be very or extremely knowledgeable), and creating a healthy bedtime routine (46.2% reported to be very or extremely knowledgeable). Participants reported to be least knowledgeable about muscle- and bone-strengthening activities (71.8% reported to be not at all or somewhat knowledgeable), fundamental movement skills (54.9% reported to be not at all or somewhat knowledgeable), and how to limit sedentary behaviours at home (47.1% reported to be not at all or somewhat knowledgeable). See Table 2 for full ratings of participants’ perceived knowledge.

**Table 2:** Parents’ perceived knowledge of physical activity, sedentary behaviour, and sleep concepts in early childhood

Item	Not at all Knowledgeable *N* (%)	Somewhat Knowledgeable *N* (%)	Knowledgeable *N* (%)	Very Knowledgeable *N* (%)	Extremely Knowledgeable *N* (%)
Fundamental Movement Skills	55 (10.2)	240 (44.7)	150 (27.9)	73 (13.6)	19 (3.5)
Muscle and Bone-Strengthening Activities	157 (29.3)	228 (42.5)	97 (18.1)	40 (7.5)	14 (2.6)
Health Benefits of Physical Activity	11 (2.1)	94 (17.5)	210 (39.2)	155 (28.9)	66 (12.3)
Health Risks of Excessive Sedentary Behaviour	23 (4.3)	138 (25.7)	197 (36.8)	132 (24.6)	46 (8.6)
Health Risks of Excessive Screen Time	17 (3.2)	150 (28.0)	182 (34.0)	127 (23.7)	59 (11.0)
How to Minimize Sedentary Behaviours at Home	54 (10.1)	198 (37.0)	167 (31.2)	86 (16.1)	30 (5.6)
How to Limit Screen Time at Home	28 (5.2)	170 (31.7)	176 (32.8)	110 (20.5)	52 (9.7)
Creating a Healthy Bedtime Routine	10 (1.9)	89 (16.6)	190 (35.4)	173 (32.2)	75 (14.0)
Safe Sleep Practices	3 (0.6)	22 (4.1)	158 (29.5)	219 (40.9)	134 (25.0)
Risky Play	34 (6.3)	148 (27.6)	188 (35.0)	129 (24.0)	38 (7.1)
Health Benefits of Outdoor Play	5 (0.9)	66 (12.3)	196 (36.6)	169 (31.6)	99 (18.5)

Regression analyses indicated that only participants’ highest level of education (*p* = .004) and whether they reported to achieve the physical activity guideline within the *Canadian 24-Hour Movement Guidelines for Adults* (*p* < .001) were significantly related to their perceived knowledge of movement behaviour concepts in early childhood (Table 1). Participants whose highest level of education was graduate school reported significantly higher perceived knowledge compared to participants whose highest level of education was high school (MD = 6.18, 95% CI = 0.90, 11.45, *p* = .022) or college (MD = 5.59, 95% CI = 2.18, 8.99, *p* = .001). Additionally, participants with an undergraduate university degree reported significantly higher levels of perceived knowledge compared to participants with a college degree (MD = 4.48, 95% CI = 1.24, 7.71, *p* = .007). Moreover, participants who reported to adhere to the physical activity guideline exhibited significantly higher ratings of perceived knowledge compared to those who did not adhere to the guidelines (MD = 3.81, 95% CI = 1.70, 5.91, *p* < .001.). A detailed description of results can be seen in Appendix C.

### Self-efficacy

Overall, parents were moderately confident with regard to movement behaviours in early childhood (*M* = 6.01; *SD* = 1.73). Parents/guardians reported the highest levels of self-efficacy for facilitating physical activity opportunities for their child(ren) everyday (*M* = 7.55; *SD* = 2.19), supporting their child(ren) in meeting age-appropriate sleep recommendations (*M* = 7.03; *SD* = 2.11), and providing their child(ren) with outdoor play opportunities everyday (*M* = 6.77; *SD* = 2.27). Parents/guardians reported the lowest levels of self-efficacy for serving as a positive role model for their child(ren)’s screen behaviours (*M* = 4.37; *SD* = 2.34) and their child(ren)’s sedentary behaviours (*M* = 5.07; *SD* = 2.39). See Table 3 for full ratings of participants’ self-efficacy.

**Table 3:** Parents’ self-efficacy relating to physical activity, sedentary behaviour, and sleep concepts in early childhood

Item	*N*	M (SD)
Facilitate physical activity opportunities for my child(ren) everyday	526	7.55 (2.19)
Lead activities that promote my child(ren)’s development of fundamental movement skills	526	6.17 (2.28)
Teach my children about the health benefits of physical activity	525	6.38 (2.26)
Serve as a positive role model for my child(ren)’s physical activity by participating in movement-based activities	526	5.91 (2.45)
Serve as a positive role model for my child(ren)’s sedentary behaviours by limiting my own sitting	526	5.07 (2.39)
Serve as a positive role model for my child(ren)’s screen behaviours by limiting my own screen use	526	4.37 (2.34)
Minimize long periods of sitting time (> 60 min) among my child(ren)	526	5.89 (2.28)
Adhere to age-appropriate recommendations for screen time among my child(ren) (i.e., no screen time < 2 years, maximum 1 h/day 2–4 years)	523	5.26 (2.87)
Engage my child(ren) in age-appropriate risky play (i.e., adventurous play that tests children’s limits such as playing at heights or high speeds)	524	5.78 (2.37)
Provide my child(ren) with outdoor play opportunities everyday	523	6.77 (2.27)
Support my child(ren) in meeting age-appropriate sleep recommendations (i.e., 14–17 h/day < 4 months; 12–16 h/day 4–11 months; 11–14 h/day 1–2 years; 10–13 h/day 3–4 years)	523	7.03 (2.11)

Results from the regression model demonstrated that participants’ ethnicity (*p* = .028) and employment status (*p* = .023) were significantly related to perceptions of self-efficacy (Table 1). Specifically, participants who identified as White reported significantly higher levels of self-efficacy compared to participants from minority ethnic groups (MD = 5.45, 95% CI = 0.60, 10.29, *p* = .028). Further, participants who were employed part-time reported significantly lower levels of self-efficacy compared to participants who were employed full-time (MD = -9.28, 95% CI = -16.31, -2.26, *p* = .010) and those employed in occasional/support positions (MD = -21.21, 95% CI = -35.83, -6.58, *p* = .005). Additionally, participants employed in occasional/support positions reported significantly greater self-efficacy than participants on parental leave (MD = 15.03 95% CI = 1.15, 28.92, *p* = .034). Lastly, participants who reported to adhere to the physical activity guideline within the *Canadian 24-Hour Movement Guidelines for Adults* showed significantly higher ratings of self-efficacy compared to participants who did not achieve the guideline (MD = 15.20, 95% CI = 10.54, 19.85, *p* < .001). No other independent variable examined had a significant relationship with self-efficacy. Complete results from the regression models are displayed in Appendix C.

## Discussion

This study aimed to explore parents’ and guardians’ knowledge of the *Canadian 24-Hour Movement Behaviour Guidelines for the Early Years*, and their perceived knowledge and self-efficacy of movement behaviour concepts in early childhood. Findings showed that parents were moderately knowledgeable and confident about movement behaviour concepts; however, their perceptions varied between specific movement behaviours and practices. This study also examined if sociodemographic characteristics, parents’ personal physical activity and screen time habits, or the movement behaviour education they received in their prenatal, postnatal, or pediatric care were related to parents’ knowledge and self-efficacy. Results demonstrated that parents of higher socioeconomic status, and parents who reported to be more physically active, displayed higher ratings across knowledge and self-efficacy outcomes. These results highlight areas of improvement to better support parents/guardians in raising healthy active children from infancy, including important equity considerations, and are discussed below.

In general, both awareness of the existence of the *Canadian 24-Hour Movement Guidelines for the Early Years* and recall of specific components of the guidelines were poor. While some age-specific recommendations were correctly estimated by parents (e.g., sleep recommendations, infant screen time limit), participants were largely unfamiliar with the physical activity recommendations and the screen time recommendation for children 2 to 4 years old. This is consistent with qualitative research conducted by Riazi and colleagues [32] who found that most parents (*n* = 40) in their focus groups were not aware of the physical activity or sedentary behaviour recommendations, and that only a few recalled some information about sleep. Evidently, current dissemination efforts for the Canadian guidelines are not reaching end users, and more targeted delivery is needed. As suggested by parents and educators in Riazi and colleagues’ study [32], medical settings, childcare centres, and community centres might represent the best avenues for delivery, with a particular emphasis on pediatrician/doctor visits due to the profession’s alignment with healthy child development, as well as the frequency that parents visit these settings for their child’s publicly funded immunizations. As reported in Bruijns et al. [28], parents and guardians in the present study rarely relied on their child’s physician for movement behaviour information, but this may be due to these behaviours going unmentioned by physicians during appointments. If more deliberate attention is paid by medical professionals to sharing information about the guidelines with their patients, parents in Canada might acquire a greater understanding about what a healthy 24 h looks like and be more inclined to go to their child’s physician for support in achieving these recommendations.

While ensuring parents and guardians are familiar with daily movement behaviour recommendations their child should achieve is important, having knowledge about a variety of movement behaviour concepts in early childhood is perhaps even more essential for practical everyday utility. In this study, participants reported to be most knowledgeable about sleep concepts (e.g., safe sleep practices, creating a healthy bedtime routine) and the health benefits of outdoor play, and the least knowledgeable about physical activity (e.g., muscle- and bone-strengthening activities, fundamental movement skills) and sedentary behaviour concepts (e.g., how to limit sedentary behaviours at home) in early childhood. This trend echoes parents’ familiarity with guidelines, with most parents reporting sufficient knowledge about their child’s sleep, but not their child’s physical activity or sedentary behaviour. Identifying parents’ knowledge gaps is important to help inform educational interventions for this group. A prime example of the positive impact of such interventions is the Melbourne Infant Feeding and Nutrition Trial (INFANT), a community-based obesity prevention intervention in Australia, aiming to improve parenting skills to support the development of a healthy diet and movement behaviours [33]. In a longitudinal analysis of this program with 292 mother/infant pairs, researchers found strong evidence that there were sustained positive effects of the INFANT program on maternal television viewing knowledge at both the 2- and 3.5-year follow-ups, and that greater knowledge among mothers was associated with less television viewing time among their children at both time points [34]. With many parents and guardians in Canada communicating their desire to learn more about movement behaviours in early childhood [28], adapting the INFANT program to the Canadian context might be a viable capacity building opportunity.

With less than 35% of parents and guardians reporting to have received sedentary behaviour information during their prenatal, postnatal, or pediatric care visits [28], it is not surprising that sedentary behaviour-related self-efficacy items, inclusive of time spent sitting/restrained and time engaged in screen-viewing, were the lowest rated among participants. Interestingly, the lowest-rated sedentary behaviour and physical activity items were tied to parents’ self-efficacy to role model healthy movement behaviours. A review by Hesketh and colleagues [35] synthesized the qualitative literature relating to barriers and facilitators to movement behaviours in young children (< 6 years) and highlighted that while parents acknowledged their role in modelling healthy movement behaviours, barriers such as lack of time and energy, and having to juggle multiple schedules, often inhibited this. This may be problematic, given the dyadic associations between parents’ and children’s movement behaviours. In fact, in a nationally representative sample of 1,116 preschoolers (3–5 years) in Canada, Carson et al. [36] found that parental physical activity and sedentary behaviours were significantly correlated with children’s movement behaviours. As such, increased support for parents and guardians, including strategies to effectively and efficiently role model healthy movement behaviours, is needed to help them feel confident in their ability to positively influence their child’s physical activity and sedentary behaviour habits.

Given the established link between parents’ and children’s movement behaviours [36] it was not surprising that parents and guardians who reported meeting the physical activity guideline within the *Canadian 24-Hour Movement Guidelines for Adults* [31] were both more knowledgeable and confident in promoting healthy movement behaviours for their children than those who were less active. This trend has also been demonstrated in studies with early childhood educators; among a sample of 417 educators, Bruijns and colleagues [37] found that those who reported achieving physical activity guideline were significantly less likely to report low self-efficacy to facilitate healthy movement behaviours for young children compared to those not achieving the guideline (*p* < .05). Considering parental self-efficacy has been noted as a significant predictor for children’s physical activity [24], efforts to improve parents’ physical activity levels should be considered as a method to increase their perceptions of self-efficacy relating to movement behaviours. As demonstrated by Kieslinger et al. [24] doing so could result in substantial benefits for children; they found that if parental self-efficacy was improved by one unit at baseline, the odds of higher physical activity levels among children increased by 39% at follow-up. The important influence that parents have on their children’s movement behaviours is clear, and the innate link to their own physical activity levels, their self-efficacy to provide movement affordances, and role modelling these important behaviours, are all important considerations for future interventions.

In addition to participants’ physical activity levels, several other sociodemographic variables were associated with parents’/guardians’ knowledge and self-efficacy. First, parents/guardians in this study with higher education reported higher perceived knowledge of movement behaviour concepts than those with lower levels of education. This stands to reason, as Lee and colleagues [38] found that among a sample of low-income mothers (*n* = 186) in the United States, maternal health literacy showed a significant positive relationship with level of education (*r* = .33, *p* < .01). It can be postulated that parents in this study with university-level post-secondary education possess greater skills to seek out health information; in fact, Stormacq, den Broucke, and Wasinski [39] reported in their integrative review that educational attainment was the most important determinant of health literacy. Another interesting finding from the multivariate analyses was that parents/guardians who identified as White reported higher levels of self-efficacy than those from minority ethnic groups. While previous research has found ethnicity to be a significant predictor of maternal self-efficacy in the early postnatal period [40], to our knowledge, this is the first study to demonstrate this association as it pertains to movement behaviour-related parenting self-efficacy. With parental employment status and family situation also demonstrating associations with knowledge and self-efficacy outcomes, it is evident that health equity is a pervasive concern that places parents and guardians with lower socioeconomic status at a clear disadvantage when it comes to promoting healthy movement behaviours among their children.

### Research implications and future directions

With obvious gaps in movement behaviour-related knowledge and self-efficacy identified among parents/guardians in this study, as well as clear sociodemographic associations with these outcomes, increased support for parents in Canada is needed to help foster healthy trajectories for young children. Specifically, improvements to the dissemination of the *Canadian 24-Hour Movement Guidelines for the Early Years* to end users (e.g., via medical and childcare settings) are needed to facilitate increased awareness of recommendations for healthy movement behaviours. Raising such awareness among parents, particularly via family physicians and trustworthy resources, may encourage parents to ask more questions to health professionals about what changes they can make to help their child(ren) achieve these standards. In addition to this, increased educational efforts are needed to ensure parents/guardians are well equipped with the knowledge needed to promote healthy movement behaviours in the home environment; according to the SCT, having a solid knowledge base will elicit improved self-efficacy to carry out the desired behaviours. This can be achieved via greater integration of movement behaviour information in primary care settings (e.g., prenatal, postnatal, or pediatric care), public health initiatives, or via training interventions led by researchers. Regardless of the setting/context, increased attention must be paid to ensuring educational programs are accessible and tailored to reach parents/guardians of lower socioeconomic status and with varied health literacy skills (e.g., offering resources at an appropriate reading level and in a variety of languages). Furthermore, future interventional research should look to explore the impacts of educational programs for parents on their movement behaviour knowledge and self-efficacy, and how sociodemographic variables (and their intersections) might influence effectiveness. Gathering qualitative insights into the barriers and facilitators to low-socioeconomic status parents’ facilitation of physical activity in the home environment would also inform the creation of educational interventions for this population.

### Strengths and limitations

This study has a number of strengths, including the size, the geographical and ethnic distribution of the sample, and the multivariate exploration of the influence of a wide range of sociodemographic variables on parental movement behaviour knowledge and self-efficacy. Yet, some limitations must be considered. First, the perceived knowledge and self-efficacy tools were exploratory in nature and, as such, were not validated. While internal consistency was good across both scales (0.92 for both perceived knowledge and self-efficacy), and the self-efficacy items were pulled from a previously validated tool for early childhood educators, cautious interpretation is needed. Second, while this study had good ethnic and geographic representation compared to the general population in Canada, the majority of participants were female, from double-parent households, highly educated, and had middle-to-upper class household incomes, which may limit generalizability to other populations.

## Conclusion

As the first study to gather a broad understanding of parents’ and guardians’ movement behaviour-related knowledge and self-efficacy as it pertains to early childhood in Canada, as well as the influence of sociodemographic factors on these outcomes, targeted educational efforts can now be made to fill noted gaps. Specifically, initiatives to raise awareness of the 24-hour movement guidelines among parents are needed to ensure they are aware of benchmarks to facilitate healthy child development. Further, increased educational opportunities, such as information delivered via healthcare providers, parenting associations, and research interventions, would help address some of the gaps in parents’/guardians’ knowledge and self-efficacy, including fundamental movement skill development and role modelling healthy sedentary behaviours. Additionally, specific attention should be paid to ensuring educational initiatives cater to the needs of parents from lower socioeconomic status groups, as this study highlighted that inequities in parental movement behaviour knowledge and self-efficacy are present. With emerging research indicating the importance of early childhood for healthy movement behaviour habit formation, targeting children’s primary role models is a good place to start.

## Electronic supplementary material

Below is the link to the electronic supplementary material.

Supplementary Material 1

## Data Availability

No datasets were generated or analysed during the current study.

## Abbreviations

INFANT: Infant Feeding and Nutrition Trial
ME & YOU: Movement Education for parents of YOUng children
SCT: Social Cognitive Theory
