# Supplementary material for: Physical activity, sedentary behaviour, and sleep knowledge and self-efficacy among parents of young children in Canada
Source: J Act Sedentary Sleep Behav. 2024 May 17;3:12. doi: 10.1186/s44167-024-00051-x (PMC11101324; doi:10.1186/s44167-024-00051-x)
Supplement: Supplementary file 1 — Supplementary Material 1 [file 44167_2024_51_MOESM1_ESM.docx]

Appendix A: ME & YOU Needs Assessment Survey

Q1 In your (or your partner's) **prenatal care**, did you receive any information regarding ***infant*** (<1y) physical activity (e.g., tummy time, fundamental movement skill development), sedentary behaviour (e.g., time restrained in a highchair/stroller/carseat, screen time), and/or sleep (e.g., recommended length of nighttime sleep and daytime naps)? Check all that apply.

- Yes - physical activity
- Yes - sedentary behaviour
- Yes - sleep
- No - I did not receive any information regarding any of the above categories
- I (or my partner) did not receive prenatal care (e.g., adoption, legal guardianship)

Q2 In your (or your partner's) **postpartum care**, did you receive any information regarding ***infant*** (<1y) physical activity (e.g., tummy time, fundamental movement skill development), sedentary behaviour (e.g., time restrained in a highchair/stroller/carseat, screen time), and/or sleep (e.g., recommended length of nighttime sleep and daytime naps)? Check all that apply.

- Yes - physical activity
- Yes - sedentary behaviour
- Yes - sleep
- No - I did not receive any information regarding any of the above categories
- I (or my partner) did not receive postpartum care (e.g., adoption, legal guardianship)

Q3 During your child(ren)'s **pediatrician/doctor appointments**, did you receive any information regarding healthy physical activity (e.g., tummy time, fundamental movement skill development, energetic play), sedentary behaviour (e.g., time sitting or restrained in a highchair/stroller/carseat, screen time), and/or sleep (e.g., recommended length of nighttime sleep and daytime naps) behaviours in early childhood (<5 years)? Check all that apply.

- Yes - physical activity
- Yes - sedentary behaviour
- Yes - sleep
- No - I did not receive any information regarding any of the above topics
- We have not had any pediatrician/doctor appointments

Q4 Where do you get information about your child(ren)'s physical activity, sedentary behaviour, or sleep? Check all that apply.

- Internet websites and news articles (If yes, which sites?) __________________________________________________
- Social media (If yes, which platform?) __________________________________________________
- Apps (If yes, which apps?) __________________________________________________
- My child's pediatrician/doctor
- Research studies
- Family and/or friends
- Other (please describe): __________________________________________________
- I do not source information about any of these topics

| Page Break |  |
| --- | --- |

Q5 Are you familiar with the ***Canadian 24-Hour Movement Guidelines for the Early Years (0-4 years)***?

- Yes
- No

Skip To: End of Block If Are you familiar with the Canadian 24-Hour Movement Guidelines for the Early Years (0-4 years)? = No

Q6 How familiar are you with the ***Canadian 24-Hour Movement Guidelines for the Early Years (0-4 years)***?

- Somewhat familiar
- Familiar
- Very Familiar
- Extremely familiar

Q7 Where did you hear about the ***Canadian 24-Hour Movement Guidelines for the Early Years (0-4 years)***?

________________________________________________________________

End of Block: Movement Behaviour Training and Education

Start of Block: Knowledge

Q8 The following questions will test your knowledge of the **Canadian 24-Hour Movement Guidelines for the Early Years (0-4 years).***If you don't know the answer, select "not sure" rather than guessing***:**

Q9 How many minutes of *tummy time* are **infants (<1 year)** recommended to engage in each day?

- 10 minutes
- 20 minutes
- 30 minutes
- 40 minutes
- Not sure

Q10 How many minutes of *total physical activity* (i.e., any intensity physical activity) are **toddlers (1-2 years) and preschoolers (3-4 years)** recommended to engage in each day?

- 60 minutes
- 90 minutes
- 120 minutes
- 180 minutes
- Not sure

Q11 How many minutes of *moderate-to-vigorous physical activity* (i.e., higher intensity physical activity) are **preschoolers (3-4 years)** recommended to engage in each day?

- 30 minutes
- 60 minutes
- 90 minutes
- 120 minutes
- Not sure

Q12 How many minutes of *screen time* should a **1-year-old** be limited to each day?

- 0 minutes
- 30 minutes
- 60 minutes
- 90 minutes
- Not sure

Q13 How many minutes of *screen time* should a **3-year-old** be limited to each day?

- 0 minutes
- 30 minutes
- 60 minutes
- 90 minutes
- Not sure

Q14 How much *good-quality sleep*, including naps, should **infants (4-11 months)** get each day?

- 10-13 hours
- 11-14 hours
- 12-16 hours
- 14-17 hours
- Not sure

Q15 How much *good-quality sleep*, including naps, should toddlers (1-2 years) get each day?

- 10-13 hours
- 11-14 hours
- 12-16 hours
- 14-17 hours
- Not sure

Q16 How much *good-quality sleep*, including naps, should preschoolers (and 3-4 years) get each day?

- 10-13 hours
- 11-14 hours
- 12-16 hours
- 14-17 hours
- Not sure

Q17 Please use the following scale to rate your perceived knowledge of ***common topics related to physical activity, sedentary behaviour, sleep, and outdoor play***among young children:

|  | Not at all knowledgeable | Somewhat knowledgeable | Knowledgeable | Very knowledgeable | Extremely knowledgeable |
| --- | --- | --- | --- | --- | --- |
| Fundamental movement skill (e.g., jumping, throwing, balancing) development |  |  |  |  |  |
| Muscle- and bone-strengthening activities |  |  |  |  |  |
| Health benefits of physical activity |  |  |  |  |  |
| Health risks of excessive sedentary (i.e., sitting/reclining) behaviour |  |  |  |  |  |
| Health risks of excessive screen time |  |  |  |  |  |
| How to minimize sedentary behaviours at home |  |  |  |  |  |
| How to limit screen time at home |  |  |  |  |  |
| Creating a healthy bedtime routine |  |  |  |  |  |
| Safe sleep practices |  |  |  |  |  |
| Risky play (i.e., adventurous play that tests children's limits but may involve a minor chance of injury) |  |  |  |  |  |
| Health benefits of outdoor play |  |  |  |  |  |

End of Block: Knowledge

Q18 Please indicate, on a scale from 0 (not confident at all) to 10 (completely confident), **how confident you are in your ability to do the following during a typical day**:

|  | 0  Not confident  at all | 1 | 2 | 3 | 4 | 5 Moderately confident | 6 | 7 | 8 | 9 | 10  Completely confident |
| --- | --- | --- | --- | --- | --- | --- | --- | --- | --- | --- | --- |
| Facilitate *physical activity* opportunities for my child(ren) **everyday** |  |  |  |  |  |  |  |  |  |  |  |
| Lead activities that promote my child(ren)’s development of **fundamental movement skills** (e.g., including running, jumping, balancing, throwing, catching) |  |  |  |  |  |  |  |  |  |  |  |
| Teach my child(ren) about the health benefits of physical activity |  |  |  |  |  |  |  |  |  |  |  |
| Serve as a positive role model for my child(ren)’s **physical activity** by participating in movement-based activities |  |  |  |  |  |  |  |  |  |  |  |
| Serve as a positive role model for my child(ren)’s **sedentary behaviours** by limiting my own sitting |  |  |  |  |  |  |  |  |  |  |  |
| Serve as a positive role model for my child(ren)’s **screen behaviours** by limiting my own screen use |  |  |  |  |  |  |  |  |  |  |  |
| Minimize long periods of **sitting time** (>60 minutes) among my children |  |  |  |  |  |  |  |  |  |  |  |
| Adhere to age-appropriate recommendations for **screen time** among my children (i.e., no screen time <2 years, maximum 1hr/day 2-4 years) |  |  |  |  |  |  |  |  |  |  |  |
| Engage my child(ren) in age-appropriate **risky play** (i.e., adventurous play that tests children's limits such as playing at heights or high speeds) |  |  |  |  |  |  |  |  |  |  |  |
| Provide my child(ren) with outdoor play opportunities **everyday** |  |  |  |  |  |  |  |  |  |  |  |
| Support my child(ren) in meeting **age-appropriate sleep** recommendations (i.e., 14-17hrs/day <4 months; 12-16hrs/day 4-11 months; 11-14hrs/day 1-2 years; 10-13hrs/day 3-4 years) |  |  |  |  |  |  |  |  |  |  |  |

Q19 Which **physical activity** topics, if any, would you like (or would you have liked) to receive more information and/or resources about? (Check all that apply)

- Gross motor development (e.g., rolling over, sitting up, standing, walking)
- Fine motor development (e.g., pincer grasp, using cutlery, drawing)
- Fundamental movement skills (e.g., running, jumping, throwing, catching)
- Tummy time
- Energetic (i.e., higher intensity) play
- Muscle- and bone-strengthening activities (e.g., climbing, jumping)
- Structured (adult-led) physical activity ideas
- Unstructured (child-led) active play
- Facilitating indoor active play
- Other (please describe): __________________________________________________

Q20 Which **sedentary behaviour** topics, if any, would you like (or would you have liked) to receive more information and/or resources about? (Check all that apply)

- Prolonged sitting time
- Prolonged time spent restrained in a highchair/car seat/stroller
- Age-appropriate screen time recommendations
- Activity ideas to break up sitting time
- Incorporating movement into traditionally sedentary activities (e.g., reading, crafts)
- Other (please describe): __________________________________________________

Q21 Which **sleep** topics, if any, would you like (or would you have liked) to receive more information and/or resources about? (Check all that apply)

- Age-appropriate sleep recommendations for nighttime and daytime sleep
- Wake windows
- Safe sleep recommendations (i.e., to prevent Spontaneous Infant Death Syndrome)
- Co-sleeping
- Independent sleep
- Sleepy cues
- Other (please describe): __________________________________________________

Q22 Which **outdoor play** topics, if any, would you like (or would you have liked) to receive more information and/or resources about? (Check all that apply)

- Health benefits of outdoor play
- How to promote outdoor free play at home
- How to dress my child(ren) for outdoor play in all weather
- Outdoor activity examples in all types of weather
- How to support my child(ren)'s outdoor risky play
- How to design an outdoor play area to support my child(ren)'s physical activity
- Other (please describe): __________________________________________________

Q23 Which format would you prefer for receiving information about your child(ren)'s physical activity, sedentary behaviour, and/or sleep? (Check all that apply)

- Online platform (i.e., website with a login)
- Social media (e.g., Instagram, Facebook)
- App
- Email
- Text messages
- Paper resource package
- Online resource package (i.e., PDF)
- Other (please describe): __________________________________________________

Q24 Please state your age:______

Q25 What gender do you identify with? (Refers to **current gender** which may be different from sex assigned at birth and may be different from what is indicated on legal documents).

- Male
- Female
- Prefer not to answer
- Prefer to self-describe: __________________________________________________

Q26 What is your racial background/ethnicity? (If you identify with more than one of the options below, you may choose to select one of these or simply specify your mixed racial background in the "Prefer to self-describe" text box)

- Arab
- Black
- White (i.e., Caucasian)
- Indigenous Peoples of Canada
- Indigenous (outside of Canada)
- Latin, Central, or South American (e.g., Brazilian, Chilean, Columbian, Mexican)
- East Asian (e.g., Chinese, Japanese, Korean, Taiwanese)
- South Asian (e.g., Indian, Pakistani, Sri Lankan)
- Southeast Asian (e.g., Cambodian, Indonesian, Laotian, Vietnamese)
- West Asian (e.g., Afghan, Iranian, Syrian)
- Prefer to self-describe __________________________________________________
- Prefer not to answer

Display This Question:

If What is your racial background/ethnicity? (If you identify with more than one of the options belo... = Indigenous Peoples of Canada

Q27 The Canadian Census identifies the following categories in its Census of the Population. Which Canadian Indigenous group do you identify with the most?

- First Nations
- Inuit
- Métis
- I prefer not to answer

Q28 What province/territory do you live in?

Select province/territory (1)

▼ British Columbia (1) ... Prince Edward Island (9)

Q29 What is your family situation?

- Single-parent
- Double-parent
- Guardian-led
- Other: __________________________________________________
- Prefer not to answer

Q30 How many children (any age) do you currently provide care for?

________________________________________________________________

Q31 How many children do you have in each of the below age categories?

|  | 0 | 1 | 2 | 3 | 4 | 5 | 6 | 7 | 8 | 9 | 10 |
| --- | --- | --- | --- | --- | --- | --- | --- | --- | --- | --- | --- |

| Infant (<1 year) () | 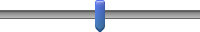 |
| --- | --- |
| Toddler (1-2 years) () | 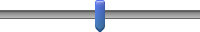 |
| Preschooler (3-4 years) () | 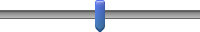 |

Q32 What type of care is your child/are your children in? (If you use a mixed care arrangement, please describe in the "Other" text box)

- Parental care
- Centre-based childcare or preschool
- Home-based (i.e., family) childcare
- Full-day kindergarten
- Other (please describe): __________________________________________________

Q33 How many **hours per week** do you spend in **moderate-to-vigorous physical activity** (e.g., jogging, running, weight lifting, playing sports)?

- >1 hour/week
- 1 to 1.4 hours/week
- 1.5 to 1.9 hours/week
- 2 to 2.4 hours/week
- 2.5+ hours/week

Q34 How many **hours of recreational screen time** (e.g., personal use of phone, tablet, computer, and/or television) do you engage in **each day**?

- >1 hour/day
- 1 to 1.9 hours/day
- 2 to 2.9 hours/day
- 3+ hours/day

Q35 What is your highest level of education?

- High school
- College
- University
- Graduate school
- Prefer not to answer

Q36 What is your current employment status?

- Full-time
- Part-time
- Occasional/Support
- Unemployed
- On parental leave from full-time employment
- On short or long-term disability leave from full-time employment
- Prefer not to answer
- Other (please describe): __________________________________________________

Q37 What is your approximate **yearly total household income (before taxes)**?

- Less than $20,000
- $20,000 - $39,000
- $40,000 - $ 59,000
- $60,000 - $79,000
- $80,000 - $99,000
- $100,000 - $119,000
- $120,000 - $139,000
- More than $140,000
- Prefer not to answer

Q38 In what housing type do you live?

- Apartment
- Condominium
- Townhouse
- Semi-detached house
- Detached house
- Other housing? Please describe: __________________________________________________

**Appendix B: Participant Demographics for Multivariate Regression Analysis Sample**

| Appendix B. *Participant Demographics for Multivariate Regression Analysis Sample (n = 350)* | |
| --- | --- |
|  | N (%) |
| Age *M* (*SD*) | 34.52 (3.82) |
| Sex  Male  Female | 22 (6.3%)  328 (93.7%) |
| Ethnicity  White  Ethnic minority^a^ | 268 (76.6%)  82 (23.4%) |
| Province/Territory  British Columbia  Alberta  Saskatchewan  Manitoba  Ontario  Québec  Maritimes and Territories^b^ | 57 (16.3%)  45 (12.9%)  28 (8.0%)  30 (8.6%)  123 (35.1%)  25 (7.1%)  42 (12.0%) |
| Family situation  Single parent  Double parent | 11 (3.1%)  339 (96.9%) |
| Number of children caring for  One  Two  Three or more | 190 (54.3%)  123 (35.1%)  37 (10.6%) |
| Highest level of education  High School  College  University  Graduate school | 12 (3.4%)  36 (10.3%)  173 (49.4%)  129 (36.9%) |
| Employment status  Full-time  Part-time  Parental leave  Occasional/support  Unemployed  Other | 146 (41.7)  35 (10.0)  120 (34.3)  9 (2.6%)  20 (5.7%)  20 (5.7%) |
| Annual household income  Less than $40,000  $40,000 to $79,000  $80,000 to $119,000  More than $120,000 | 18 (5.1%)  39 (11.1%)  89 (25.4%)  204 (58.3%) |
| Housing type  Apartment  Condominium  Townhouse  Semi-detached house  Detached house  Other | 24 (6.9%)  22 (6.3%)  42 (12.0%)  22 (6.3%)  228 (65.1%)  12 (3.4%) |
| Meeting physical activity guideline | 81 (23.1%) |
| Meeting screen-time guideline | 229 (65.4%) |
| Survey language  English  French | 337 (96.3%)  13 (3.7%) |
| Received prenatal physical activity education | 151 (43.1%) |
| Received prenatal sedentary education | 103 (29.4% |
| Received prenatal sleep education | 117 (33.4%) |
| Received postnatal physical activity education | 214 (61.1%) |
| Received postnatal sedentary education | 123 (35.1%) |
| Received postnatal sleep education | 159 (45.4%) |
| Received pediatric physical activity education | 193 (55.1%) |
| Received pediatric sedentary education | 92 (26.3%) |
| Received pediatric sleep education | 114 (32.6%) |

**Appendix C: Detailed Results from the Regression Models Testing Associations with Self-efficacy, Perceived Knowledge, and Knowledge of Canadian 24-Hour Movement Guidelines for the Early Years**

| Appendix C. *Detailed Results from the Regression Models Testing Associations with Self-efficacy, Perceived Knowledge, and Knowledge of Canadian 24-Hour Movement Guidelines for the Early Years* | | | | | | | | | |
| --- | --- | --- | --- | --- | --- | --- | --- | --- | --- |
|  | Self-Efficacy | | | Perceived Knowledge | | | Knowledge of Guidelines | | |
|  | *b* | *se* | *p* | *b* | *se* | *p* | *b* | *se* | *p* |
| Age | -0.18 | 0.28 | .537 | -0.19 | 0.13 | .134 | -0.01 | 0.03 | .668 |
| Sex  Male  Female | REF  1.71 | 4.49 | .703 | REF  0.37 | 2.05 | .856 | REF  0.55 | 0.44 | .212 |
| Ethnicity  White  Ethnic minority^a^ | REF  -5.45 | 2.46 | .028* | REF  -0.74 | 1.13 | .514 | REF  -0.06 | 0.25 | .820 |
| Province/Territory  British Columbia  Alberta  Saskatchewan  Manitoba  Ontario  Québec  Maritimes and Territories^b^ | REF  -2.39  -6.22  -4.09  -2.89  -0.19  -1.30 | 3.87  4.37  4.37  3.11  5.99  4.00 | .538  .156  .350  .353  .974  .746 | REF  -0.97  -0.57  1.54  -0.70  1.27  0.74 | 1.76  1.99  1.97  1.41  2.73  1.82 | .582  .775  .436  .613  .642  .687 | REF  -0.51  -0.79  -0.34  -0.51  0.46  -0.74 | 0.29  0.44  0.43  0.31  0.60  0.40 | .192  .072  .433  .102  .444  .067 |
| Family situation  Single parent  Double parent | REF  -2.23 | 3.87 | .727 | REF  4.36 | 2.89 | .133 | REF  1.42 | 0.64 | .027 |
| Number of children cared for  One  Two  Three or more | REF  -1.36  -3.12 | 2.26  3.51 | .550  .375 | REF  -0.59  1.02 | 1.03  1.59 | .566  .522 | REF  0.42  0.16 | 0.23  0.35 | .062  .651 |
| Highest level of education  High School  College  University  Graduate school | REF  -4.17  -1.37  -0.15 | 6.28  5.70  5.90 | .980  .811  .507 | REF  0.89  5.06  6.18 | 2.86  2.59  2.68 | .837  .051  .022* | REF  -0.14  -0.47  -0.31 | 0.63  0.57  0.59 | .821  .416  .821 |
| Employment status  Full-time  Part-time  Parental leave  Occasional/support  Unemployed  Other | REF  -9.28  -3.11  11.93  -0.80  4.24 | 3.56  2.43  7.00  4.92  4.52 | .010*  .203  .089  .871  .349 | REF  -3.61  -2.37  2.06  -3.75  0.23 | 1.67  1.10  3.18  2.24  2.05 | .031  .033  .517  .094  .911 | REF  -0.25  -0.82  0.67  -0.57  0.09 | 0.36  0.24  0.70  0.49  0.45 | .481  <.001*  .340  .251  .839 |
| Annual household income  Less than $40,000  $40,000 to $79,000  $80,000 to $119,000  More than $120,000 | REF  -5.51  -1.55  2.78 | 5.88  5.80  5.87 | .349  .789  .636 | REF  -0.06  -2.50  -0.76 | 2.67  2.64  2.67 | .982  .344  .775 | REF  -0.59  -0.17  0.20 | 0.59  0.58  0.59 | .321  .775  .733 |
| Housing type  Apartment  Condominium  Townhouse  Semi-detached house  Detached house  Other | REF  -16.68  -11.82  -12.45  -9.88  -11.39 | 5.62  4.99  5.64  4.32  6.53 | .003  .019  .028  .023  .082 | REF  -3.34  -3.91  -1.86  -2.58  -4.62 | 2.54  2.27  2.62  1.97  2.97 | .189  .086  .478  .191  .121 | REF  -0.48  -0.57  -0.42  -0.27  -0.49 | 0.56  0.50  0.57  0.43  0.66 | .389  .255  .463  .540  .452 |
| Meeting physical activity guideline | 15.20 | 2.36 | <.001* | 3.81 | 1.07 | <.001* | -0.07 | 0.24 | .757 |
| Meeting screen-time guideline | 3.59 | 2.08 | .085 | -0.53 | 0.95 | .575 | 0.12 | 0.21 | .578 |
| Survey language  English  French | REF  -1.59 | 7.36 | .829 | REF  -1.10 | 3.42 | .747 | REF  -0.04 | 0.74 | .959 |
| Received prenatal physical activity education | 2.08 | 2.94 | .479 | 0.31 | 1.35 | .817 | 0.32 | 0.30 | .287 |
| Received prenatal sedentary education | -1.54 | 3.41 | .652 | -0.61 | 1.57 | .697 | 0.36 | 0.34 | .291 |
| Received prenatal sleep education | 0.54 | 3.16 | .864 | -0.22 | 1.45 | .882 | -0.51 | 0.32 | .108 |
| Received postnatal physical activity education | -1.07 | 2.53 | .673 | -1.51 | 1.15 | .190 | 0.19 | 0.25 | .453 |
| Received postnatal sedentary education | -1.41 | 2.72 | .604 | -0.19 | 1.24 | .691 | -0.53 | 0.27 | .052 |
| Received postnatal sleep education | 3.44 | 2.64 | .193 | 0.28 | 1.20 | .815 | -0.06 | 0.27 | .829 |
| Received pediatric physical activity education | 2.38 | 2.41 | .323 | .14 | 1.10 | .896 | -0.12 | 0.24 | .625 |
| Received pediatric sedentary education | 1.72 | 2.94 | .561 | -1.20 | 1.34 | .371 | 0.41 | 0.29 | .166 |
| Received pediatric sleep education | -0.72 | 2.72 | .791 | -0.93 | 1.25 | .457 | 0.11 | 0.27 | .675 |
| *Note*. ^a^ = ethnic minority includes all ethnicity choices that were not ‘White/Caucasian’; ^b^ = Maritimes and Territories includes New Brunswick, Nova Scotia, Prince Edward Island, Newfoundland and Labrador, Nunavut, Northwest Territories, and Yukon; * = *p* < .05. | | | | | | | | | |
